# Parents’ experiences with a home-based upper limb training program using a video coaching approach for infants and toddlers with unilateral cerebral palsy: a qualitative interview study

**DOI:** 10.1186/s12887-022-03432-w

**Published:** 2022-06-29

**Authors:** Anke PM Verhaegh, Nienke B Nuijen, Pauline BM Aarts, Maria W G Nijhuis-van der Sanden, Michèl AAP Willemsen, Brenda E Groen, Johanna E Vriezekolk

**Affiliations:** 1 Department of Pediatric Rehabilitation, Sint Maartenskliniek, Nijmegen, The Netherlands; 2 IQ Healthcare, Research Institute for Health Sciences, Radboud University Medical Center, Nijmegen, The Netherlands; 3 Department of Research and Innovation, Sint Maartenskliniek, Nijmegen, The Netherlands; 4 Department of Rehabilitation, Donders Institute for Brain, Cognition and Behavior, Radboud University Medical Center, Nijmegen, The Netherlands; 5 Department of Pediatric Neurology, Amalia Children’s Hospital, Radboud University Medical Center, Nijmegen, The Netherlands; 6 Donders Institute for Brain, Cognition and Behavior, Radboud University Medical Center, Nijmegen, The Netherlands

**Keywords:** Early intervention, Unilateral cerebral palsy, Home training program, Video coaching, Occupational therapy

## Abstract

**Background:**

Although early home-based upper limb training programs are promising, in-depth understanding of parents’ experiences with these programs is still limited. We developed an early home-based upper limb training program for infants and toddlers (8–36 months) with or at risk of unilateral cerebral palsy using video coaching for parents. The aim of this qualitative study was to evaluate parents’ experiences with the home-based training program using a video coaching approach in order to optimize implementation strategies.

**Methods:**

We held semi-structured interviews with parents of 13 children with unilateral cerebral palsy, who participated in our program in the period from 2014 – 2017. On average, parents had delivered two training periods of the program at the time of the interviews. Interviews were analyzed using inductive thematic content analysis.

**Results:**

We identified three overarching interacting themes that shaped the experiences of parents with the program: 1) Parental learning comprising the subthemes parents’ training competencies and the facilitative and reinforcing role of video coaching, 2) Parental load comprising the subthemes flexibility of the program, supportive network, competing demands, and child’s mood and functional capacities, and 3) Parental perseverance comprising the subthemes beliefs and expectancies and seeing child’s functional improvements.

**Conclusions:**

For successful implementation of an early home-based upper limb training program using video coaching, support in delivering home-training from a therapist or from others within parents’ social network, is needed to relieve parental load. Seeing functional improvements of their child on the videos increased parents’ motivation to continue with the training. Positively phrased feedback from an occupational therapist stimulated parents’ perseverance and training competency.

**Supplementary Information:**

The online version contains supplementary material available at 10.1186/s12887-022-03432-w.

## Background

Cerebral palsy (CP) is the most common childhood physical disability, with a worldwide prevalence of 2.1 per 1000 live births [1]. CP comprises a group of permanent developmental disorders caused by damage or malformation of the developing fetal or infant brain [2]. Unilateral CP accounts for about 40% of children with CP [3]. Children with unilateral CP have motor function impairments on one side of the body [4]. Many children with unilateral CP disregard the use of the affected upper limb when they, already in early development, discover it is more efficient to perform activities with the non-affected upper limb [5, 6]. If children with unilateral CP do not learn how to use the affected upper limb, this may lead to reduced hand skill development, a deficit in bimanual task performance, and diminished participation in daily life [7]. To improve the use of the affected upper limb in children with unilateral CP, Constraint Induced Movement Therapy (CIMT) and Bimanual Training (BiT) are effective interventions provided that the dose of therapy is sufficient [5] and that the therapy preferably starts at a young age [8].

Home-based programs have been introduced as a useful strategy for increasing training dose in rehabilitation practice for children with CP [9]. In home-based programs, the child performs therapeutic activities with parental assistance in the home environment with the support and coaching of a therapist [9]. Home-based programs for children and infants are promising [9–11]. Parents are key in the delivery of home-based therapy programs. Parents of 5 to 12-year-old children with CP reported that home-based programs were more time-efficient than attending hospital-based programs and that the programs enhanced their competence in how to help their child [12]. Coaching and follow-up support from a therapist at regular intervals were identified as important by parents [12]. Yet, difficulties with incorporating a home-based program in daily life routines [13] and finding enough time for the training in busy family life [14] have also been reported. In-depth understanding of parents’ experiences with intensive upper limb home-based training programs for very young children remains limited, but it is important to consider these experiences when determining the effectiveness of implementation of these programs. We had previously developed and implemented an early home-based CIMT and BiT training program using a novel video coaching approach for parents of infants and toddlers (8–36 months of age) with or at risk of unilateral CP, to allow parents and children not living nearby our rehabilitation center to participate in our program. A video coaching approach may be less intrusive for family life and more flexible than home visits by the therapist. The aim of this qualitative study was to evaluate parents’ experiences with the home-based training program using a video coaching approach in order to optimize implementation strategies.

## Methods

### Research design and setting

This study used a qualitative research design with face-to-face semi-structured interviews with parents of children who participated in our early home-based upper limb training program in the period from 2014–2017. This program was designed for infants and toddlers of 8–36 months of age with or at high risk of unilateral CP. The training program consisted of an eight-week period of intensive upper limb training (30 min daily, seven days per week) delivered by the parents at home. Remote video coaching by the occupational therapist of our rehabilitation center supported parents to deliver the training to their child at home. Coaching entailed predominantly written feedback and suggestions. Besides the training program, the child received usual care (primary care pediatric physical therapy). The training program and video coaching approach is described in more detail in Additional file 1.

### Participants

Through purposive sampling [15], all 16 parents of children with unilateral CP who had participated in our early home-based upper limb training program until December 2017 were invited for this study. In January 2018, the child’s occupational therapist of the Sint Maartenskliniek informed all parents about the study by phone, email, or face-to-face at the rehabilitation center. All parents received written information about the study. Of the original 16 parents, three did not respond and 13 parents gave informed consent and participated in the interviews. In all cases except one, the mother was interviewed. On average, parents had delivered two training periods of the program at the time of the interviews. All parents had sufficient knowledge of the Dutch language. Table 1 presents the characteristics of the 13 participating parents and their children.

**Table 1:** Characteristics of the participating parents and their child

Participant	Parent				Child		
	Interviewee	Highest level of education^b^	Training periods^c^	Number of children	Sex	Age (corrected in months)^d^	Mini-MACS classification^e^
1	Father	Tertiary	4	1	Female	9	3
2	Mother	Tertiary	2	5	Male	33	3
3	Mother	Tertiary	1	3	Female	25	1
4	Mother	Tertiary	1	1	Male	23^a^	2
5	Mother	Secondary	2	2 (twins)	Male	11^a^	2
6	Mother	Tertiary	3	1	Male	12	3
7	Mother	Tertiary	2	3	Male	11	4
8	Mother	Secondary	1	1	Male	20^a^	3
9	Mother	Tertiary	1	2	Female	21	4
10	Mother	Secondary	2	2	Male	19	3
11	Mother	Tertiary	3	3	Male	10	3
12	Mother	Tertiary	1	2	Male	26	1
13	Mother	-	3	3	Female	20	3

^a^Born prematurely

^b^Highest level of education: The UNESCO International Standard Classification of Education (2011) was used

^c^Experience with conducting the home training program at the time of the interview; 1 period = 8 weeks

^d^Age at the start of the first home training program

^e^*Mini-MACS* Mini Manual Ability Classification System. Classification was established at the age of 1 year, or when entering the program

The ethical review board of the University Medical Centre Nijmegen exempted the study (protocol reference number: 2017/3998) from ethical approval according to the Dutch Medical Research Involving Humans Acts. Written informed consent was obtained from all parents prior to the interview and data were reported anonymously. All methods were carried out in accordance with relevant guidelines and standards and the Standards for Reporting Qualitative Research guideline was used to ensure complete and transparent reporting [16].

### Interviews

All interviews were conducted between January and March 2018 by one researcher (NN, master student Biomedical Sciences), at the location of the parents’ preference (their homes or the rehabilitation center). The researcher (NN) was trained in interviewing techniques using instruction videos; she had no clinical background and was not involved in the treatment of the children. The interviews were held with the parent who was involved in most of the home training sessions with the child. The interviews were audio-recorded and additional field notes were made during and after the interviews. The semi-structured interview guide consisted of one lead question “Can you tell me about your experiences with the program?”, followed by five main theme questions [17] specific to the research aim: “What do you think of the feasibility of the program?”, “What do you think of the guidance you received during the program?”, “How did you experience the video coaching you received during the program?”, “What were the main benefits of participating in the program?”, and “What were your expectations of the program?”. In addition, probing questions (“Can you tell me more?”, “Can you give me an example?”) were asked to gain more details. All questions were open-ended to yield rich data [17, 18]. 

### Data analysis

Data collection and data analysis were alternated and repetitively reflected upon by three researchers (NN, AV, and JV). Data were analyzed following the principles of inductive thematic content analysis [19]. The following steps were taken: familiarization with the data, initial coding (assigning relevant text fragments to codes) and refinement of coding, searching for (sub)themes, reviewing and defining of (sub)themes, and identification of relationships between themes, and selection of quotations. Interviews were anonymized by giving each participant a number. Initial data analyses were executed by two researchers, NN and AV. The latter was an experienced (> 10 years of experience in working with children with CP) occupational therapist (MSc) with expertise in CIMT and BiT interventions and trained in qualitative research techniques. As an occupational therapist, she was involved in coaching some of the participating families in this study.

For familiarization with the data, the interviews were transcribed verbatim by two researchers (NN, AV), read and re-read, and initial ideas were noted down on memos. During the initial coding phase, all transcripts were read thoroughly and initial codes were assigned to relevant text fragments. One researcher (NN) transcribed and generated initial codes for 8 of the 13 interviews. To ensure trustworthiness, the initial codes generated from four of these latter transcripts were compared to the initial codes independently generated by the second researcher (AV). The remaining five interviews were transcribed and analyzed by the second researcher (AV). The two researchers (NN, AV) continuously and repetitively reflected on and refined these initial codes taking field notes into account. In the next phase of searching for themes, relevant codes were grouped into themes and sub-themes, and some initial ideas (including those written down on memos) about the relationship between themes and sub-themes were generated through reflexive dialogue [19]. To review the themes and sub-themes, three team meetings were held between researchers and health professionals with diverse expertise. The team included the two researchers (NN, AV), and researchers (MN, PA, BG, JV) with clinical and research expertise in the field of pediatric physical therapy, pediatric occupational therapy, and health psychology. They discussed, rephrased, and reordered the themes and sub-themes, taking into account the quotes identified by the two researchers (NN, AV) until consensus was reached. In the final phase, quotations were selected by the team members to illustrate the themes, and relationships between themes and subthemes were presented in an integrative conceptual model. Data saturation was reached when no new (sub)themes could be identified in the last two interviews. The software program ATLAS.ti was used for the coding process.

## Results

Ten interviews were held at the homes of the parents, and three in a private room at the rehabilitation center. The duration of the interviews ranged from 45 to 70 min (mean 56.5 min, SD = 9.4 min).

Overall, parents were positive about the program's home-based character and found practicing with their child for 30 min a day was demanding, but doable. They enjoyed being able to practice with their child themselves in their home environment. Most parents were positive about the opportunity to conduct the training (with professional therapeutic coaching) in the earliest possible phase of their child’s upper limb motor development. Though, many parents described the program as *‘very intense’*, and *‘exhausting’* for themselves and sometimes also for their child. Almost all parents noted that it was hard work practicing daily for eight consecutive weeks; they reported having underestimated the time and energy it costs to deliver the program.

**Fig. 1:**
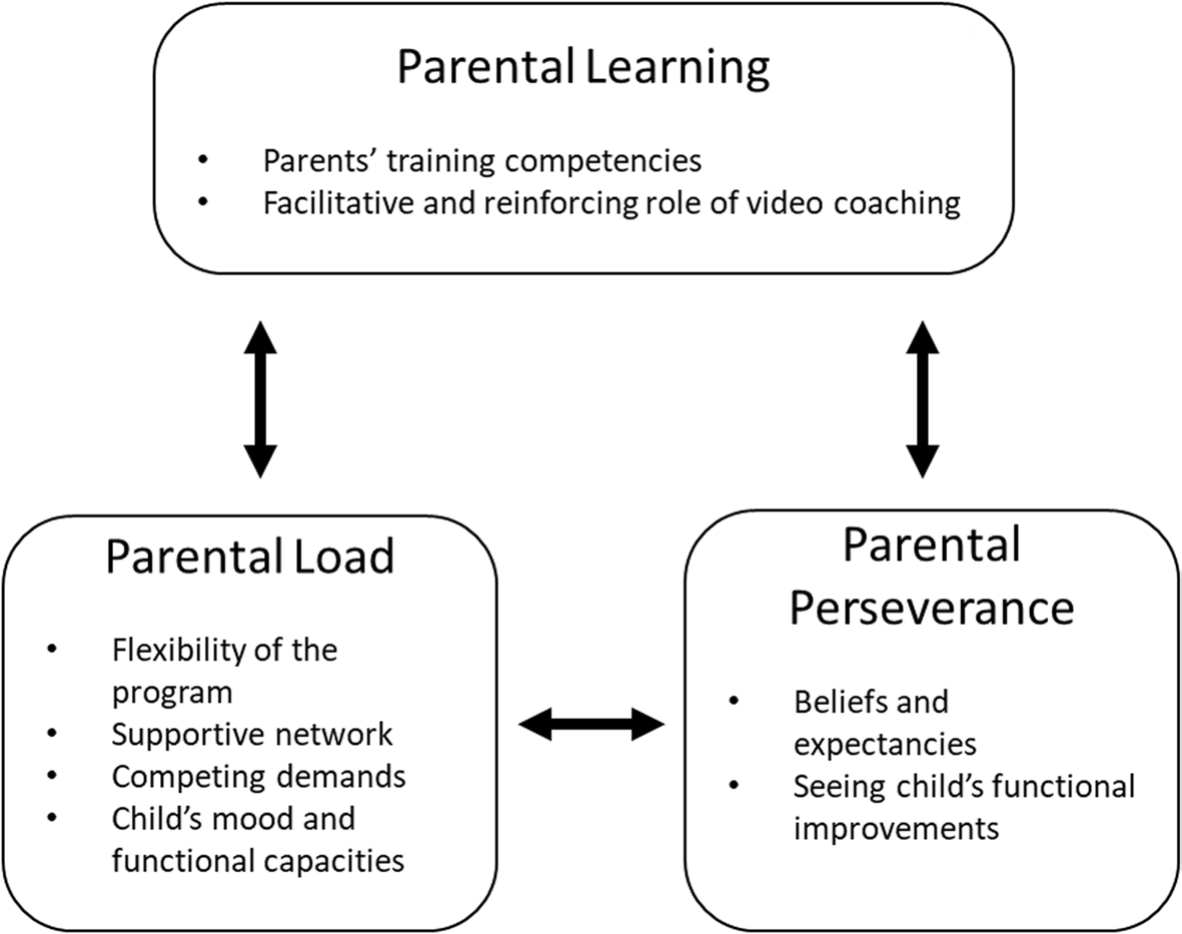
Conceptual model reflecting the interrelatedness between themes identified relevant for implementation of an early home-based upper limb training program using video coaching

Three overarching interacting themes aided our understanding of parents’ experiences with the home-based program with video-coaching: Parental learning, Parental load, and Parental perseverance. Parents gradually became a skilled training provider; this parental learning process was influenced by the perceived load of being a training provider and parents’ perseverance in the training program (Fig. 1).

### Theme: Parental learning

As the training program progressed parents acquired the competencies (e.g., planning the training in a busy daily life, being creative) to successfully deliver the program. Provision of remote video coaching facilitated and reinforced the parents to provide the training.

#### Parents’ training competencies

Parents felt capable enough to provide the training themselves, though, in the beginning, it took more effort. Some parents mentioned that providing the training became easier with the second or even third training block. All parents felt that by delivering the training themselves they became increasingly aware of their child’s capacities and learned how to stimulate their child in using and expanding these capacities in the affected limb.

*“(…) I realized that I had to hand things to him differently, cause otherwise, it’s really easy to just give it to him on the good side. So I also put his food on the other side, on the difficult side instead of the easy side. So I also started approaching him differently myself (…) Before, I didn’t stimulate that other side at all, and through the CIMT I did start doing that. So that was also a learning process for me (…).”- Parent 10*

Some parents reported that the need for coaching decreased when the program progressed.

*“Well, to start with, we recorded many videos, (…) to get a feel of what we were meant to be doing and how we wanted to do it. And the last two times, we haven’t sent in as many anymore because, well, it was clear what [child’s name] needed to do and what she liked to do.” - Parent 1*

After the program, stimulating the affected upper limb became part of parents’ daily parenting routines. The awareness of training opportunities in daily life situations was felt as a major benefit of the program, irrespective of the improvements their child had made.

*“Yes, it has actually just become part of our parenting. Yeah, because after those eight weeks we didn’t stop mind you, I just continued doing it.”- Parent 4*

One of the challenges was to fit the training into their daily lives and to adapt their daily routines to be able to completely focus on their child for 30 min. Some parents felt having a fixed training schedule made it easier.

*“I had a routine going when I did it. I did it [the training] every morning with fruit, and I tried to do it in the afternoon at the table with a sweetie or something. Cause if I didn’t have that [routine], then the day would just fly by and then I wouldn’t have done it.”- Parent 10*

Another main challenge parents mentioned was being creative as a training provider, i.e. coming up with various play activities to keep their child engaged. Almost all parents reported that their child lost interest at some point when no new toys or activities were offered. Parents reported that the box with toys they received at the start of the program made it possible to start practicing straight away without having to collect appropriate toys themselves. Receiving this box with toys gave parents some initial ideas of play that elicited the targeted movements.

*“They’d explained what was in the box and what you could do with it. We also gave it our own twist (..) we had something at home that he really liked and we thought it was a perfect fit, but it wasn’t in the box. Let’s go ahead and use that as well.”- Parent 8*

#### Facilitative and reinforcing role of video coaching

Overall parents were positive about the idea of sharing video-recordings of the training sessions to receive coaching and to communicate with the occupational therapist at the rehabilitation center. Because parents could decide what parts of the training session to record and share, they were assured that the occupational therapist would give feedback on those parts of the training that parents felt were important. To be able to show how the home training went instead of trying to explain verbally was considered as a huge advantage.

*“You know, the advantage of a video is, like during the weekends I have more time, you can see the good things, but also the things that went wrong, so you can ask yourself ‘what should I do with this?’. You can ask specific questions (…), … otherwise it would only just be a snapshot of that one time a week or whenever they [occupational therapist] come by.”- Parent 8*

Almost all parents expressed that they needed coaching to know how to work on their child’s training goals, how to approach their child, and for inspiration for which toys and activities to use in the training session. It was important for parents to get informed whether the occupational therapist noticed an improvement in the hand function of their child and they appreciated the confirmation when they were on the right track or how they could adjust the training. Parents stated that it was very helpful to get a quick and very enthusiastic and positive response to the video-recordings of the training session they uploaded. This motivated and stimulated them to continue with the program.

*“So yeah, we actually always got a quick response, elaborate and very enthusiastic (…) we also just really liked reading it every time, cause every time we thought: oh yes, he’s doing really well! And well, that motivates you to continue.”- Parent 6*

### Theme: Parental load

Delivering the program to the child meant that parents had to find a balance between their roles as a parent and training provider. The flexibility of the program as well as a supportive network reduced the parental load, whereas other features of the program (e.g., high training intensity, making and uploading video registrations), parents’ competing demands, and their child’s limited functional capacities increased the parental load.

#### Flexibility of the program

The main benefit of the program was its flexibility. Not having to travel to the rehabilitation center on multiple scheduled times a week was practical and fitted parents’ busy family schedule and, in turn, had less impact on the other children in the family.

*“I think it’s great that we could just do that in our own time and in our own way (…) with no other people around, (...) no other appointments to attend either and you know, you do have a small child that still needs to have a nap, as well as two brothers who need to be picked up from school (…)” - Parent 11*

The flexibility of the program also made it possible for parents to decide the best moment for the child to provide the training session; their child had to be wide-awake and in a good mood for the training session to work out.

*“Yes, I actually found that very pleasant, being in your own environment: at home, he [the child] could decide when [he would practice] and otherwise that moment would be planned [with a therapist], and then it’d be the question whether he was up to it.”- Parent 4*

#### Supportive network

Many parents received some help in delivering the training, for example from the grandparents, the primary care pediatric physical therapist or the daycare center. Since the child then received the training session at appropriate moments during working days, these parents perceived the training intensity as less problematic.

*“And that’s what I liked about this, you have, you get more of a feeling that you are really all doing it together, and so, you all think: right, this is it; we are going to get this done for the next eight weeks.” - Parent 11*

Most parents felt supported by the primary care pediatric physical therapist and mentioned it was an advantage that the pediatric physical therapist had known the child and the family for a long time. Parents were relieved that the pediatric physical therapist practiced with their child once or multiple times per week replacing a parent’s training session. Some pediatric physical therapists practiced at the child's daycare center which enabled parents to reach the required training intensity on their working days.

*“I liked actually having a physical therapist as well, and that they came along every week. That did help me because, on that day, the day of their visit, I could leave things to the physical therapist for a while.” - Parent 3*

#### Competing demands

Practicing with the child on working days was challenging because the child was often too tired to practice after a day at daycare. One parent reported feelings of guilt when she could not practice with her child because of the child being too tired. On non-working days, parents could plan the sessions on times best suitable for the child or split the sessions into a few shorter intervals (e.g. three 10-min sessions). One parent mentioned it was easier for her to make it fun when she was relaxed and had enough time. On workdays, she perceived this as far more difficult.

*“When I’d pick him up from the daycare at half past 5 or quarter to 6, and then he’d still need to do something he finds very difficult for another half hour, and something that just costs him a huge amount of energy, then, of course, he’ll really resist.”- Parent 6*

Parents mentioned having other children at home during the training sessions brought challenges in dividing their attention between the children; the training session took their full attention and effort to keep a very young child focused even during a ten-minute play session. In contrast, some parents mentioned involving brothers or sisters in the play session was very stimulating for the child.

*“Sometimes the other children could be a bother, but on the other hand sometimes they’d even help her (…) especially when she [the child] wasn’t motivated, then I just sat her brother next to her, and let him do the exercises, and then she copied him.”- Parent 3*

Parents also mentioned difficulties with providing the training and recording the video at the same time. Therefore, they had to plan a moment together with their partner.

*“I literally had my hands full just doing the exercises, and so I was unable to record a video as well, or I just couldn’t do it (…) so I mostly only made recordings when there were two of us (…).” - Parent 3*

Some parents felt making video-recordings was a time-consuming task. They perceived it as an extra burden to the already high training intensity, especially when technical problems arose (e.g. slow internet connections, the Video Question Box website not working properly or the smartphone’s storage capacity being full).

*“I found that quite a time-consuming task as well, by the way. (…) So indeed you’ve then trained for half an hour, and then you have to film somewhere in between (…). And then having to upload everything, that always kept us occupied for a whole evening per week, and then also having to type up that story of what you wanted to know, how are things going? So I thought that was quite intense, that was something on top of everything else.”- Parent 6*

#### Child’s mood and functional capacities

Parents of children with very limited functional capacities of the affected upper limb felt that it remained challenging to provide the training. Particularly when their child could cognitively cope with more difficult games than their functional capacities allowed.

*“Yes he was already one year old then, but his [motor] skills were like being at a baby level…, hitting toward and reaching for things a bit, it was quite hard to find toys that he liked.” - Parent 6*

Parents mentioned that integrating the training into daily routines would get easier when the child grew older and the functional capacities of the affected hand improved.

“We also did it during cooking, for example, then we’d put [name child] near the sink giving him beans, washing them under the tap, that sort of thing.”- Parent 11

Parents stated that the child’s enjoyment during practicing was very important to keep their child engaged during the training. Few parents reported their child did not enjoy practicing which in turn also affected their own enjoyment and adherence to the program.

“Ehm, and if he had enjoyed it all, for all of the eight weeks, we would have taken part and enjoyed it much more; and also kept going for eight weeks.”- Parent 8

### Theme: Parental perseverance

Parents’ beliefs and expectancies towards the home-based program and the observed improvements of their child’s upper limb performance influenced parents’ perseverance in the program.

#### Beliefs and expectancies

The main drive for parents’ program participation was that they felt they could make a difference for their child by getting the most out of it. Parents considered the commitment to the program and the willingness to invest time to reach high training intensity key to success. Some parents strongly believed in a positive effect of practicing despite not always seeing an improvement in the child’s upper limb function straight away. Believing they contributed to their child’s future stimulated them to go on with the program.

*“Well yes, if you ask me, I did really believe that lots of things were happening that I could not yet see. And so, that many things were happening in that little head that would deliver some favorable results in the long term.”- Parent 7*

Some parents felt pressure having to practice with their child on a daily basis, but this pressure was not always interpreted as being negative as parents’ felt that some pressure helped to adhere to the program. Framing the training sessions with their child as ‘playtime’ helped their engagement in the program.

*“You can see it as exercising, but you can also just see it as a game at the time, so I think it’s also about how you look at it. I think I saw it too much as ‘having to’ then.”- Parent 12*

As emphasized by some parents, the training intensity was felt as a key element of the program’s success. Knowing that the training block would end after eight weeks of training, stimulated parents to persevere until the break.

*“So you just know: we’re going to give it our all those 8 weeks (…) And.. then 8 weeks seem doable (...) then you know that there’s a beginning and an end to it and then you have another 8 weeks rest, and we always really enjoyed that you know (…). And that also helped, so after 8 weeks you felt, well go ahead, here we go again.”- Parent 6*

#### Seeing child’s functional improvements

Through the uploaded video clips, parents tracked the improvements in their child’s upper limb function performance, which motivated the parents to continue the program.

*“Well, about the video question box, what’s nice about it, is that you can always see everything listed, I did really like that, and also that earlier programs were saved, (…). So you just quickly look back again and think: oh yes, that’s what it was like then. And then you immediately see the real mega difference of course (…) it really gives you a type of diary in a way.” - Parent 11*

Other parents found the commitment to the program challenging. Not seeing an improvement straight away confirmed their prior reluctance (e.g. anticipated resistance of their child) to the program. Some parents reported feelings of frustration when they did not notice improvements after several weeks of practice.

*“And with much pain and effort, he started to use his hand to push something off the table but that’s really as far as it went. So, ehm, yes, that was really very frustrating after all of that time we had spent on it.”- Parent 7*

## Discussion

In this interview study, we evaluated parents’ experiences with our early home-based upper limb training program for infants and toddlers with unilateral CP using a video coaching approach. These experiences yielded a conceptual model reflecting three overarching interacting themes; Parental learning, Parental load and Parental perseverance relevant for optimizing implementation strategies for home-based therapy programs using video coaching. In the following sections, the most important findings will be discussed.

Being a training provider led to parents’ enhanced competence, knowledge and awareness of opportunities to stimulate their child’s upper limb function. Some parents even indicated that providing the home-based program themselves was the greatest benefit because they felt educated even beyond the program’s scope. Parents reported that providing the training took more effort at the beginning of the program and required more feedback from the therapist than later on. Video coaching enabled therapists to be easily accessible and to give timely, flexible support adapted to parents’ needs. Expecting parents to provide training on a daily basis leads to the need for a quick and positively phrased response of the therapist, especially when parents struggle with providing the training. Previously it has been shown that program adherence increases by twofold when a therapist frequently evaluates caregiver skills and possible difficulties during the training program [20]. A main challenge parents experienced as a training provider was being creative to come up with various play activities to enhance motivation and enjoyment, especially during the last weeks of the training block. Although parents received tips from therapists this may not be enough to provide parents with resources (e.g. variations of toys and play activities at just the right training level) to enable parents to keep training sessions fun. These observations are in line with other studies [9, 21]. To facilitate parents’ need for creativity, a list of activities and toys matching the child-specific goals may be useful to offer to the parents similar as in the study of Smidt et al. [14]. Additionally, an (online) platform could be installed that enables the exchange of experiences and suggestions between parents for toys and tasks that could be integrated into training sessions.

In line with previous research [22], our study showed that being a parent and a training provider puts a significant demand on parents' daily lives as they must balance the home training program with their normal everyday activities. The availability of a supportive network when carrying out the training program reduced perceived parental load. When grandparents or others from parents’ social networks or the primary care pediatric physical therapist provided some of the training sessions, parents felt, temporarily, relieved of their training provider load. Video coaching appears to be supportive as it increases the flexibility in planning the training sessions as parents do not have to travel to the rehabilitation center and no additional home visits by the therapist have to be planned. However, giving priority to the training program in busy family lives remained challenging for most parents as also reported by Smidt et al. [14], in particular when other issues (e.g. the child’s health-related issues or development of other motor milestones such as independent sitting or crawling) demanded parents’ full attention. These competing priorities could hamper providing a home-based program focusing on upper limb development. Therefore, as suggested by others [22], an in-depth needs inventory prior to the start of the intervention is imperative to identify these possible competing priorities. To fit the program into busy family lives, the training schedule should be individualized, and as also reported previously [20, 23]. When the perceived parental load was high, for example on working days, it was difficult to provide an enjoyable training. Parents consistently expressed that it is crucial that the child enjoys the training. When their child did not, this also had an impact on parents’ enjoyment, motivation, and effort to provide the training session. As parents seem to underestimate the time and energy it can take to provide the training, therapists should monitor parental load during the home-based program [24]. Consistent with the findings of others [21, 25] technical problems were perceived as time-consuming and could add to the perceived parental load of training.

Parental motivation is a key factor for successful implementation of the home-based intervention. Adding a video coaching approach to our home-based program contributed to keeping the parents motivated during the whole program. Especially the possibility to observe their child’s progression over time was a motivating factor as also described by others [12, 14, 20, 26, 27] which was most relevant when only very small steps in their child’s upper limb function development were achieved. When parents framed the training as playtime instead of therapy, this had a positive influence on their perseverance in the program and their efforts in creating enjoyable training sessions.

### Strengths and limitations

This study is the first, to our knowledge, that reports on parents’ experiences as a training provider in a home-based upper limb training program using a video coaching approach for babies and toddlers with unilateral CP. A strength of our study is that the interviews were performed by one researcher who was not involved in the training program. However, our study is also subject to certain limitations. Parents’ recollections of events were retrospective, relying on their memory of the intervention period with an average of 19 months ago. Therefore, the information provided by the parents might have been biased by time. Though some parents indicated they could now better reflect on that period than they could have directly after the intervention. Mistakenly, summaries of the interviews were not sent to the interviewees for a valid member check. However, all transcripts were read independently and thoroughly by two researchers, and the identified themes and subthemes were then extensively discussed in an expert group. This group comprised of researchers and health professionals to limit the effect of interpretation bias and the chance of missing themes. Since the parents were not involved in the theme identification, it might, however, still be possible that some findings were interpreted differently by the researchers. Despite the small sample size, no new topics were raised during the later interviews which indicates data saturation had been reached. Given the fact that parents from only one rehabilitation center were included, caution should be taken when generalizing the results to different clinical settings. More research is required for this purpose.

### Implications for clinical practice

Parents often found the early home-based training program using video coaching demanding. To relieve some of their training provider load, parents may need additional support in providing the training. Providing parents with sufficient information and performing an in-depth needs-inventory is important for parents to make a well-balanced decision about participating in the program. Additionally, therapists should monitor possible increases in perceived parental load as the intervention progresses. This yields the therapist information to reflect on with the parents and the opportunity to suggest program modifications (e.g. additional help in providing the training by family members or the primary care pediatric physical therapist, changes in the planning of training sessions during the day) to meet parents’ needs.

As playfulness in training sessions is considered a very important ingredient for enjoyment of the training sessions, therapists should specifically monitor this aspect of the training. Creating playful training sessions puts high demands on parents' creativity. Giving suggestions for adaptations in play activities that challenge the child at just the right level and experiencing fun at the same time is one of the main goals of the video coaching by the therapist.

Flexibility in the timing and content of the support by the therapist, and the therapist being easily accessible for the parents seems vital in implementing a successful home-based training program. Video coaching increases therapists’ accessibility for the parents and parents’ motivation to continue with the training as the recorded videos facilitated parents to see the improvement their child made over time. However, properly functioning digital equipment is a prerequisite for successful implementation of a video coaching approach. New technologies are available and should be explored to optimize the implementation of our intervention.

## Conclusion

For successful implementation of an early home-based upper limb training program using video coaching, support in delivering home-training from a therapist or from others within parents’ social network, is needed to relieve parental load. Seeing functional improvements of their child on the videos increased parents’ motivation to continue with the training. Positively phrased feedback from an occupational therapist about the videos stimulated parents’ perseverance and training competency.

## Supplementary Information

**Additional file 1.** Description of the intervention.

## Data Availability

The dataset generated and analyzed during the current study is not publicly available due to individual privacy and the sensitive nature of the interviews. Data are however available from the corresponding author upon reasonable request and with permission of the interviewees.

## Abbreviations

CP: Cerebral palsy
CIMT: Constraint Induced Movement Therapy
BiT: Bimanual Training
mini-AHA: Mini-Assisting Hand Assessment
AHA: Assisting Hand Assessment
mini-MACS: Mini-Manual Ability Classification System

## References

[1] Oskoui M, Coutinho F, Dykeman J, Jetté N, Pringsheim T (2013). An update on the prevalence of cerebral palsy: A systematic review and meta-analysis. Dev Med Child Neurol.

[2] Rosenbaum P, Paneth N, Leviton A, Goldstein M, Bax M (2007). A report: The definition and classification of cerebral palsy April 2006. Dev Med Child Neur.

[3] Wu YW, Croen LA, Shah SJ, Newman TB, Najjar DV (2006). Cerebral palsy in a term population: Risk factors and neuroimaging findings. Pediatrics.

[4] Surveillance of Cerebral Palsy in Europe (2000). Surveillance of cerebral palsy in Europe: a collaboration of cerebral palsy surveys and registers Surveillance of Cerebral Palsy in Europe (SCPE). Dev Med Child Neurol.

[5] Hoare BJ, Wallen MA, Thorley MN, Jackman ML, Carey LM, Imms C (2019). Constraint-induced movement therapy in children with unilateral cerebral palsy. Cochrane Database Syst Rev.

[6] Zielinski IM, Jongsma MLA, Baas CM, Aarts PBM, Steenbergen B (2014). Unravelling developmental disregard in children with unilateral cerebral palsy by measuring event-related potentials during a simple and complex task. BMC Neurol.

[7] Hoare B, Greaves S (2017). Unimanual versus bimanual therapy in children with unilateral cerebral palsy: Same, same, but different. J Pediatr Rehabil Med.

[8] McIntyre S, Morgan C, Walker K, Novak I (2011). Cerebral palsy-Don’t delay. Dev Disabil Res Rev.

[9] Novak I, Berry J (2014). Home program intervention effectiveness evidence. Phys Occup Ther Pediatr.

[10] Eliasson AC, Nordstrand L, Ek L, Lennartsson F, Sjöstrand L, Tedroff K (2018). The effectiveness of Baby-CIMT in infants younger than 12 months with clinical signs of unilateral-cerebral palsy; an explorative study with randomized design. Res Dev Disabil.

[11] Chamudot R, Parush S, Rigbi A, Horovitz R, Gross-Tsur V (2018). Effectiveness of modified constraint-induced movement therapy compared with bimanual therapy home programs for infants with hemiplegia: A randomized controlled trial. Am J Occup Ther.

[12] Novak I (2011). Parent experience of implementing effective home programs. Phys Occup Ther Pediatr.

[13] Beckers LWME, Geijen MME, Kleijnen J, Rameckers EAA, Schnackers MLAP, Smeets RJEM (2020). Feasibility and effectiveness of home-based therapy programmes for children with cerebral palsy: A systematic review. BMJ Open.

[14] Smidt KB, Klevberg GL, Oftedal BF (2020). Home Programme to Improve Hand Function for Children with Bilateral Cerebral Palsy: Beneficial but Challenging. Phys Occup Ther Pediatr.

[15] Robinson OC (2014). Sampling in Interview-Based Qualitative Research: A Theoretical and Practical Guide. Qual Res Psychol.

[16] O’Brien BC, Harris IB, Beckman TJ, Reed DA, Cook DA (2014). Standards for reporting qualitative research: A synthesis of recommendations. Acad Med.

[17] Kallio H, Pietilä AM, Johnson M, Kangasniemi M (2016). Systematic methodological review: developing a framework for a qualitative semi-structured interview guide. J Adv Nurs.

[18] Turner DW (2010). Qualitative interview design: A practical guide for novice investigators. Qual Rep.

[19] Braun V, Clarke V (2006). Using thematic analysis in psychology. Qual Res Psychol.

[20] Medina-Mirapeix F, Lillo-Navarro C, Montilla-Herrador J, Gacto-Sánchez M, Franco-Sierra MA, Escolar-Reina P (2017). Predictors of parents’ adherence to home exercise programs for children with developmental disabilities, regarding both exercise frequency and duration: A survey design. Eur J Phys Rehabil Med.

[21] Beckers LWME, Smeets RJEM, de Mooij MAC, Piškur B, van der Burg JJW, Rameckers EAA (2022). Process Evaluation of Home-based Bimanual Training in Children with Unilateral Cerebral Palsy (The COAD-study): A Mixed Methods Study. Dev Neurorehabil.

[22] Hanna K, Rodger S (2002). Towards family-centred practice in paediatric occupational therapy: A review of the literature on parent-therapist collaboration. Aust Occup Ther J.

[23] Lillo-Navarro C, Medina-Mirapeix F, Escolar-Reina P, Montilla-Herrador J, Gomez-Arnaldos F, Oliveira-Sousa SL (2015). Parents of children with physical disabilities perceive that characteristics of home exercise programs and physiotherapists’ teaching styles influence adherence: A qualitative study. J Physiother.

[24] Beckers LWME, Smeets RJEM, van der Burg JJW (2021). Therapy-related stress in parents of children with a physical disability: a specific concept within the construct of parental stress. Disabil Rehabil.

[25] Stockwell K, Alabdulqader E, Jackson D, Basu A, Olivier P, Pennington L (2019). Feasibility of parent communication training with remote coaching using smartphone apps. Int J Lang Commun Disord.

[26] Lord C, Rapley T, Marcroft C, Pearse J, Basu A (2018). Determinants of parent-delivered therapy interventions in children with cerebral palsy: A qualitative synthesis and checklist. Child Care Health Dev.

[27] Phoenix M, Jack SM, Rosenbaum PL, Missiuna C (2018). A grounded theory of parents’ attendance, participation and engagement in children’s developmental rehabilitation services: Part 2. The journey to child health and happiness. Disabil Rehabil.

