# Supplementary material for: Parents’ experiences with a home-based upper limb training program using a video coaching approach for infants and toddlers with unilateral cerebral palsy: a qualitative interview study
Source: BMC Pediatr. 2022 Jun 29;22:380. doi: 10.1186/s12887-022-03432-w (PMC9245237; doi:10.1186/s12887-022-03432-w)
Supplement: Supplementary file 1 — Additional file 1. Description of the intervention. [file 12887_2022_3432_MOESM1_ESM.docx]

# A1 Additional file 1: Description of the intervention

Our home-based upper limb training program is designed for infants and toddlers of 8-36 months of age at risk of or with unilateral CP. The program is a goal directed, individualized program using a distributed intensive training model. The program is based on the guiding principles of effective partnership-based home programs (1).

In our program a hybrid form of parent training and parent coaching is applied. Parents are trained by the occupational therapist during the second visit at the rehabilitation center in how to conduct the training at home. The role of the occupational therapist shifts to coaching of the parents during the home training program, by means of a video coaching approach.

### Training period and dosage

The training program consists of eight week blocks of intensive home-based upper limb training added to the usual care the child already receives, with eight weeks of usual care in between. Based on the available evidence (2) at the time of program development and concerning feasibility aspects, the intervention dosage was set at 28 hours of training over an eight week period (seven days per week, 30 minutes per day which could be split in multiple sessions during the day).

## Procedure

### First visit: Needs inventory and baseline assessment

The first visit of the parents and the child to our rehabilitation center comprises an intake interview with the parents by the pediatric rehabilitation physician and the occupational therapist to discuss the initial needs of the parents and the child, followed by a baseline assessment ((mini-) Assisting Hand Assessment (AHA)) (3) to detect areas of difficulty in manual activities of the child. Based on this information the occupational therapist discusses the possibility of participating in a home-based training program with the parents if this meets the primary needs of parents and the child. When parents decide to participate in the program with their child, within two weeks they visit the occupational therapist in our rehabilitation center for a second time to receive instructions for the home-based program.

### Second visit: Parent training

Parents are encouraged to ask the local pediatric physical therapist (often already involved with the family), grandparents or daycare center personnel to join them during this second visit. Instruction is provided concerning the home training program. Parents receive an instruction booklet designed for this intervention, illustrated with photos of the desired movements to be elicited and seating instructions. Parents receive a box filled with toys matching the child’s specific hand function goals and developmental stage of play. All toys are demonstrated to the parents and variations of using the toys addressing multiple goals are shown. Parents are encouraged to add or remove toys to and from the box when the child needs more variation.

### Intervention

The program is provided by the parents in the home environment. Every child starts with a Baby (8-18 months of age) or Toddler (18-36 months of age) Constraint Induced Movement Therapy program in order to improve unimanual capacities of the affected upper limb. As presented in Figure A.1 all children receive an individual trajectory of follow up intervention blocks tailored to their goals, developmental stage, engagement and preferences as expressed by their parents.

**Fig A1. Flow diagram of the early upper-limb intervention program**


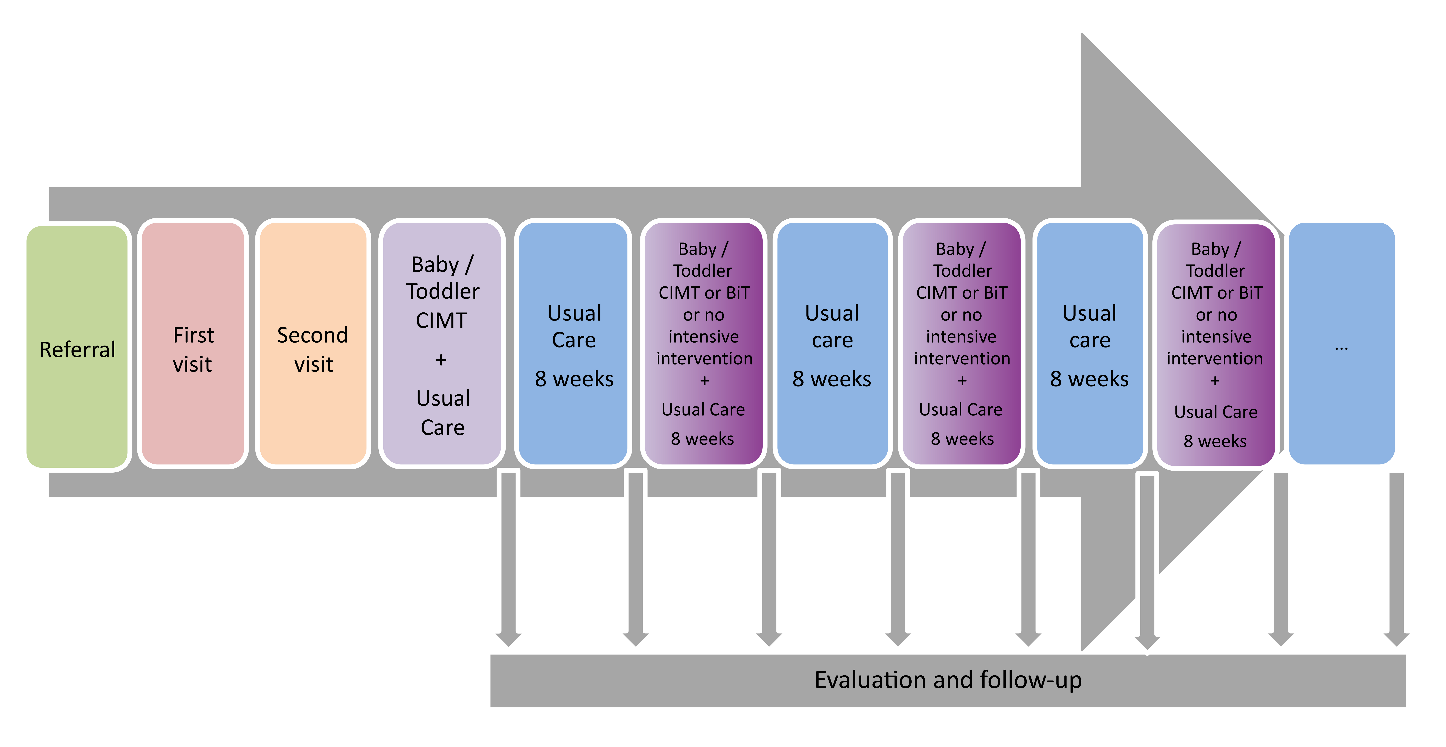


Baby or Toddler CIMT intervention block:

The Baby or Toddler CIMT intervention block aims at unimanual skill development and the program aligns with the Baby-CIMT intervention model as described by Eliasson and coworkers (4). The child wears a slipper sock with a leather sole or a sling to restrain the non-affected upper limb. Unimanual skill development is promoted by means of a high repetition rate of goal-directed actions with the affected upper limb at just the right task difficulty. The program includes components such as holding, reaching, grasping and releasing toys. A Baby or Toddler A(dvanced) variant of the program is described for children who are already able to grasp with their affected hand at the start of the intervention and the Baby or Toddler B(asic) variant of the program for children who are not (yet) able to grasp.

Baby or Toddler BiT intervention block:

Once the child improved unimanual actions through CIMT, in most cases they need to learn the strategies to guide performance of bimanual skills. Learning bimanual skills is best achieved through practice of bimanual tasks (5). The Bimanual Training in this intervention program comprises the repetitive performance of carefully chosen, goal-related, two-handed activities that elicit speciﬁc bimanual actions and behaviors as described by Hoare and Greaves (5).

### Video coaching

In order to enable parents to easily upload videos and comments on the videos with the therapists, a secure website was designed for our program, called the ‘Video Question Box’.

Parents upload their videos of the training sessions at home to the website and add written comments to the videos (i.e. remarks, questions, concerns, reflections). The therapist receives a notification of the parents’ uploaded video and their additional comments by e-mail. Then the therapist views the videos and provides the parents with written feedback on the videos in the ‘Video Question Box’. Parents then receive an e-mail notification and can now read the therapists’ feedback and suggestions.

Video coaching was provided by two occupational therapists with approximately 10 years of experience in working with children with CP. Both therapists were trained in using Motivational Interviewing techniques (6). The therapists provided regular (at least once a week) feedback and suggestions to the parents in the ‘Video Question Box’. Principles of the video coaching approach were offering information to parents (e.g. regarding hand function development, stages of play, toy and play suggestions), enabling parents to reflect on the training sessions and on the enjoyment of the child and themselves by encouraging them to watch specific sequences of the video-recordings of previous sessions, empowering parents to practice those activities matching the child’s abilities and preferences and family routines and providing emotional support to parents (by means of expressing empathy, encouraging parents through identifying and celebrating specific progress the child makes and complimenting parents actions) (7). If therapists sense it is important to speak to parents via telephone (e.g. because they noticed parents struggling with providing the training at home), they will do this.

### Usual care

Usual care in the Netherlands for infants with developmental delays is in general provided by pediatric physical therapists from private practices. The pediatric physical therapist adds (only when preferred by parents), one home-visit a week in order to remain enough time to work on gross motor developmental goals. Home visits can be dedicated to guiding parents through the implementation of the program in the home setting, answering questions and sometimes providing the training. The pediatric physical therapist collaborates with the occupational therapist from the rehabilitation center by communicating through the Video Question Box and/or by e-mail or telephone. The specific role of the pediatric physical therapist within the program mainly depends on the needs of the parents.

### Time registration

Parents fill out a time registration form on a daily base on the secure website of the ‘Video Question Box’. This enables the occupational therapist to monitor adherence and detect possible difficulties for parents in providing the training. Outcomes are discussed with the parents during the video coaching sessions and help is provided if needed.

### Evaluation and follow-up

Every eight weeks parents visit the rehabilitation center with their child in order to evaluate the outcome of the training block by means of the (mini-) AHA. Goals are evaluated and the outcome of the training from the parents’ point of view and possible follow-up of the program is discussed.

Together with the parents, a decision is made whether or not to proceed with a second intensive eight-week training program. If it is decided to proceed, a choice is made between proceeding with a Baby/ Toddler-CIMT program or a Baby/ Toddler BiT program. If it is decided not to proceed with an intensive training program, the child will be monitored by administering the (mini-) AHA at an eight-week interval rate. If later on it is still preferred to start with a training program, it can start at any time.

## References

1. Novak I, Cusick A. Home programmes in paediatric occupational therapy for children with cerebral palsy: where to start? Aust. Occup. Ther. J. 2006;53(4):251–26.
2. Lin K chung, Wang T ni, Wu C yi, Chen C ling, Chang K chieh, Lin Y chan, et al. Effects of home-based constraint-induced therapy versus dose-matched control intervention on functional outcomes and caregiver well-being in children with cerebral palsy. Res Dev Disabil. 2011; doi:10.1016/j.ridd.2011.01.023.
3. Greaves S, Imms C, Dodd K, Krumlinde-Sundholm L. Development Of The Mini-Assisting Hand Assessment: Evidence For Content And Internal Scale Validity. Dev Med Child Neurol. 2013; doi:10.1111/dmcn.12212.
4. Eliasson A-C, Sjöstrand L. Baby-CIMT Manual. 2015. https://ki.se/sites/default/files/migrate/baby-cimt_manual_20151125.pdf. Accessed 19 May 2021.
5. Hoare B, Greaves S. Unimanual versus bimanual therapy in children with unilateral cerebral palsy: Same, same, but different. J Pediatr Rehabil Med. 2017; doi: 10.3233/PRM-170410.
6. Miller W, Rollnick S. Motivational interviewing: Preparing people for change. New York, US: Guilford Press; 2002.
7. Akhbari Ziegler S, Hadders-Algra M. Coaching approaches in early intervention and paediatric rehabilitation. Dev Med Child Neurol. 2020; doi:10.1111/dmcn.14493.
